# How to evaluate the clinical outcome of joint-preserving treatment for osteonecrosis of the femoral head: development of a core outcome set

**DOI:** 10.1186/s13018-019-1364-x

**Published:** 2019-10-09

**Authors:** Zhipeng Xue, Jigao Sun, Taixian Li, Zeqing Huang, Weiheng Chen

**Affiliations:** 1 Department of Orthopaedics, China Academy of Chinese Medical Sciences, Wangjing Hospital, No. 6 Zhonghuannanlu, Chaoyang District, Beijing, 100102 China; 2 Institute of Chinese Materia Medica, China Academy of Chinese Medical Sciences, Beijing, China

**Keywords:** Osteonecrosis of the femoral head, Clinical evaluation, Core outcome set, Joint-preserving

## Abstract

**Background:**

This study aimed to develop a core outcome set (COS) for clinical trials of joint-preserving treatment for osteonecrosis of the femoral head (ONFH), that is, to define a minimal set of outcomes that should be reported in such trials.

**Methods:**

A mixed research method was adopted in this study. First, clinical trials of hip preservation therapy were systematically researched and analyzed. Second, a three-round Delphi survey involving both doctors and patients was carried out to obtain the core outcome indicators. Round 1 was a modified Delphi questionnaire for doctors and patients to determine which outcomes are important to these stakeholders, round 2 determined what clinical evaluation core outcomes should be included for the joint-preserving treatment of ONFH, and round 3 determined how core outcomes should be measured. Finally, a consensus meeting was held to discuss and vote on the established COS.

**Results:**

The results of the systematic review showed that 42 outcome indicators were classified according to common signs and symptoms, quality of life, long-term outcomes, radiological evaluation, blood biochemistry, and indexes of safety. The three rounds of Delphi surveys completed the selection of indicators for the COS and the determination of the corresponding measurements.

A total of 73 orthopedic doctors and 103 patients participated in round 1, and the top 10 indicators selected were basically the same. In round 2, 32 experts identified the following indicators: pain, range of motion (ROM) of hip flexion, walking distance, and stable rating of X-ray images. In round 3, 35 experts defined the measurement of each indicator. Finally, the consensus meeting identified the four indicators aforementioned that constituted the COS.

The scores for pain, ROM of hip flexion, and walking distance are from 0 to 10; 0 represents the best scores, while 10 represents the most serious impairment. The stable rating of X-ray images is determined by the morphology of the femoral head and the change in the density of the necrotic area.

**Conclusions:**

We established a COS for hip-preserving treatment of ONFH that includes four indicators: pain, ROM of hip flexion, walking distance, and stable rating of X-ray images.

## Background

Osteonecrosis of the femoral head (ONFH) is a progressive disabling disease that mainly affects young and middle-aged adults, and many surgeons attempt to delay performing total hip arthroplasty (THA) when making decisions [1, 2]. Therefore, several options for joint preservation have been advocated, but the indications and success rate still need to be demonstrated. This is due in large part to the lack of a standardized and widely used method of assessment [3].

Several methods or scales have been developed and used to evaluate the efficacy of interventions for the treatment of ONFH. Currently, the most commonly used methods include the Harris Hip Score (HHS) [4], the visual analogue scale (VAS) [5, 6], the Western Ontario and McMaster Universities Osteoarthritis Index (WOMAC) [7], the Charnley score [8], and the Japanese Orthopedic Association Score (JOA) [9]. Each score has its own characteristics, and some indexes are not necessarily suitable for evaluating joint preservation therapy. For example, the HHS was originally used to evaluate the efficacy of THA; now, it is widely used to evaluate various procedures [10, 11]. In the short-term, ONFH patients must use crutches to prevent femoral head collapse; however, patients who undergo THA need more autonomous walking exercises to restore function, and this may affect the joint function part of the score.

Although a wide range of randomized controlled trials (RCTs) has been published, and systematic reviews and meta-analyses have been conducted to evaluate efficacy in different settings, the reality is that insufficient attention has been paid to the outcomes in clinical trials [12, 13]. The choice of outcomes and the methods of measurement affect the analysis of data, and the impact of outcome reporting bias widely affects the conclusions of systematic reviews [14, 15].

A core outcome set (COS) defines a minimum set of outcomes to be reported in all clinical trials for a specific area that should be standardized and decided by consensus [16, 17]. It does not imply that outcomes should be restricted to those in the COS, but the COS is expected to be included and reported in all studies, and authors are encouraged to explore other outcomes. The advantages and benefits of using the COS are as follows [18]: (1) to reduce the heterogeneity among trials, (2) to simplify the design of research, and (3) to enhance the utilization of data and the quality of evidence.

A task force formed by experts was established to address issues arising from this work. The effort to develop a COS for joint preservation in ONFH included but was not limited to searching for scientific evidence in the literature, employing a modified and consensus-building Delphi method and conducting consensus meetings for verification.

## Methods

### Study design

The Core Outcome Measures in Effectiveness Trials (COMET) [17] and Core Outcome Set-STAndards for Reporting (COS-STAR) [16] guidelines were used for developing this COS. The scope of the COS was “evaluation of the effect of joint preservation treatment for ONFH”.

This study was performed in China. The medical centers included tertiary academic medical centers with a range of orthopedic, statistical, and pharmaceutical specialties. A task force was set up with researchers from 12 participating medical centers. The project was divided into two distinct phases: (1) generate an initial list of all possible outcomes (systematic review of the literature) and (2) prioritize important outcomes for different stakeholders groups by Delphi survey and verify them in consensus meetings.

### Ethical considerations

In China, official ethical approval was not required as the ethics committees confirmed that the relevant legislation was not applicable. The plan was to obtain the informed consent of the investigated personnel.

### Phase 1: Generate an initial list of all possible outcomes

#### Systematic review

This study consists of a systematic review of studies of the various interventions for joint preservation, including nonoperative therapy and operative therapy other than THA. Guidelines and consensus reports published by international clinical experts were considered to form a framework for identifying potential outcomes.

#### Types of studies

Retrieval strategies were adopted to limit the included studies to those that were high-quality and likely to influence clinical practice. Therefore, the Oxford Centre for Evidence-Based Medicine’s guidelines regarding the hierarchy of evidence were applied [19].

During the process of identifying outcomes, RCTs were given priority, and non-randomized comparative studies were considered when RCTs were lacking. It was possible that more types of study designs would identify more outcomes and would more fully reflect the views of all stakeholders. In subsequent patient interviews, the Delphi questionnaire provided ample opportunity for all stakeholders to add what they thought were potential outcomes.

The review was reported in accordance with the Preferred Reporting Items for Systematic reviews and Meta-Analyses (PRISMA) guidelines [20]. We also included other systematic reviews to identify the outcomes reported by multiple initial studies.

The primary objective of this review was to identify all possible outcomes and to finally choose the core outcomes from various types of published studies and supplement them with information obtained from Delphi questionnaires.

#### Types of interventions

Interventions considered for this review were [1, 21–23] surgery (core decompression, femoral osteotomy, nonvascularized bone grafting, vascularized bone grafting), stem cells, non-operative therapies (alendronate, lipid-lowering drugs, anticoagulants, physiotherapy, Chinese medicine therapy), and observation. We defined a time period from January 2015 to December 2018, during which we believed we could find sufficient support for the possible outcomes.

#### Exclusion criteria

Studies with participants diagnosed with adult ONFH and other complications and studies with fewer than ten patients per group were excluded because they are unlikely to influence practices.

#### Eligibility of studies

Two review authors (JGS and ZPX) independently screened the titles and abstracts of the articles obtained from the searches. The full texts of all potentially relevant studies were obtained. Any studies that did not meet the requirements were excluded. Any disagreement regarding the full texts was resolved through discussion. Where resolution was not possible, a third review author (WHC) was consulted.

#### Data extraction

Data were extracted independently by two review authors (TXL and ZQH) and checked by two other authors (ZPX and WHC) to ensure that possible outcomes were identified. The following data were extracted from each study: study type, author details, year and journal of publication, sample size, each outcome reported whether the outcome was defined, the definition used, the tools used to measure the outcome, and the time point. Disagreements were resolved by discussion.

#### Data analysis and presentation

Data were entered into Microsoft Excel for analysis. All outcomes were reported for the same or similar categories; subsequently, the categories were assessed for inclusion in the COS, and the measurement methods to be used were discussed.

#### Identification of potential outcomes

A list of possible potential outcomes (Table 1) was identified from a systematic review. The steering committee reviewed all the included results, emphasized the reduction of duplicated results for different terms, and grouped very similar results together, thus organizing all possible outcomes into different categories.

**Table 1:** List of potential outcomes

Categories	Outcomes
Common signs and symptoms	Harris Hip Score (HHS)
	Western Ontario and McMaster Universities Osteoarthritis Index (WOMAC)
	Visual analogue scale (VAS)
	Charnley score
	Merle D’Aubigne score
	Japanese Orthopedic Association Score (JOA)
	Method used to determine the percentage of adult ONFH
	Syndrome integral to TCM
	Clinical effective rate
	ROM of flexion
	Walking distance
	Degree of claudication
Quality of life	MOS Short-Form Health Survey (SF-36)
	12-item Short-Form Health Survey (SF-12)
	Quality of Life Brief Scale (QOL-BREF)
	EORTC-30 Scale
	Barthel index
Long-term outcomes	Femoral head survival time
	Hip joint arthroplasty rate
	Femoral head collapse rate
	Femoral head survival rate
Radiological evaluation	Degree of X-ray change
	Degree of computed tomography (CT) change
	Degree of bone marrow edema
	Percentage of empty lacunae
	Sample line of magnetic resonance imaging (MRI)
	Necrotic bone resorption rate
	New bone repair rate
	Bone mineral density
Blood biochemistry	Erythrocyte sedimentation rate (ESR)
	Lipid indexes
	Bone Gla protein (BGP)
	Endothelin (ET)
	Bone morphogenetic protein 2 (BMP2)
	Osteoblasts (OB)
	Osteoclasts (OC)
	Osteoprotegerin (OPG)
	Alkaline phosphatase (ALP)
	C-reactive protein (CRP)
Indexes of safety	Adverse effect rate
	Adverse events
	Detection of blood biochemical indexes

### Phase 2: Prioritize important outcomes from different stakeholders groups by Delphi survey and verify by consensus meetings

#### Modified Delphi survey

A Delphi survey of key stakeholder options was conducted to reach consensus regarding outcomes for the COS. The list of potential outcomes from phase 1 was formatted into items that could be selected by all participants and given scores on different levels for inclusion in the final COS. The items were edited and worded for both the patient group and the medical professionals group to ensure understanding. Although there is no consensus about the sample size when using the Delphi methodology, we referred to the previous Delphi survey for the COS and were guided by the COMET. It was anticipated that each Delphi process would consist of three rounds, with a total of 140 experts and 103 patients participating.

The experts were selected from various research areas in the field of orthopedics, including the treatment of ONFH with the various joint-preserving surgeries described above, comprehensive treatment with non-surgical therapies, and combined treatment with traditional Chinese and Western medicine. We consulted the opinions of the participating experts in advance, provided detailed information about the project and the questionnaire through email, and finally counted the answers.

#### Delphi survey, round 1

Web-based surveys were administered to patients and clinicians (separate surveys were conducted in parallel) to select the outcomes of importance. The participants received an introduction about the project’s objectives and were asked to choose (yes/no) whether each outcome should be included in the proposed COS for joint-preserving treatment of ONFH. The participants were given the opportunity to suggest any additional outcomes that were not provided in the presented list.

#### Analysis of round 1

We recruited 103 patients and 73 clinicians (at a ratio of 3:2) from clinics in 10 provinces in China over a 6-week period. The patients were clearly diagnosed with ONFH and were purposively selected to represent all types of ONFH patients, including varied stages of the Association Research Circulation Osseous (ARCO) classification. This round comprised 103 ONFH patients (156 hips), 39 females and 64 males, aged between 18 and 90 years, with an average age of 45.9 years. The number of patients with each ARCO classification was as follows: I: 13, II: 37, III: 73, and IV: 33. The 73 clinicians comprised 24 residents, 27 attending doctors, and 22 senior doctors. They had a range of work experience from 1 to 32 years, with an average of 11.4 years.

The analysis was conducted separately for patients and clinicians to identify discrepancies, but the results were similar (Table 2). The top five evaluation outcomes selected by both patients and clinicians were pain, joint motion, walking distance, stable rating of X-ray images, and limping.

**Table 2:** The top ten outcomes through round 1

Outcomes	103 ONFH patients		73 doctors	
	Frequency (*n*)	Percentage (%)	Frequency (*n*)	Percentage (%)
Pain	91	88	69	96
Joint motion	66	64	55	76
Walking distance	49	48	39	54
Limping	47	46	31	43
Stable rating of X-ray images	42	41	52	72
Going up and down the stairs	28	27	14	19
Joint deformities	23	22	12	17
Wearing shoes and socks	18	17	22	31
Using a cane	15	15	13	18
Sitting in a chair	14	14	7	10
Riding the bus	2	2	2	3

#### Delphi survey, round 2

The comprehensive list of potential outcomes and any additional outcomes suggested by the participants in round 1 was presented to the expert panel. Responses from the first round were disseminated anonymously to the expert panel for discussion. The participants were asked to vote (yes/no) on whether each outcome should be included in the proposed COS using this new information. For this round, the Delphi expert panel included a target sample size of 32 respondents. The outcomes chosen by more than 70% of participants in the expert panel were regarded as the core outcomes to be used.

#### Analysis of round 2

Thirty-two experts with an average of more than 10 years of clinical experience in the treatment of ONFH were selected to complete the questionnaire in this round. Pain, joint motion, walking distance, and stable rating of X-ray images were chosen by more than 70% of the participants. Therefore, these four outcome indicators were retained as the COS.

#### Delphi survey, round 3

Before this round of questionnaires was conducted, the measurement methods used to determine the outcomes of the previous round was searched systematically, and an additional 35 experts were selected and asked to determine how to measure the outcomes.

#### Analysis of round 3

All the results were shown to the experts at the conference, and 35 experts attended this round. Their initial consensus was as follows: the use of a visual analogue scale (VAS) to measure pain, the ROM of hip flexion to measure joint motion, the meters walked to measure autonomous walking, and the change in the morphology and density of the femoral head to measure the stability of X-ray images. Referring to Pennsylvania [24], ARCO stages [25], and our previous study [26], the stable rating of X-ray images was defined as follows: the necrotic area of the femoral head had a similar or the same morphology before and after treatment and the necrotic area showed that bone density had increased, the cystic size had decreased, and the sclerosis zone was blurred (Table 3).

**Table 3:** Preliminary core outcomes and measurement methods

Outcome	Measurement
Pain	Visual analogue scale (VAS)
ROM of hip flexion	Degree of flexion
Walking distance	Meters of autonomous walking
Stable rating of X-ray images	Morphological change of the femoral head and density change in the necrotic area

#### Final consensus meeting

A face-to-face consensus meeting with key stakeholders was held after the completion of the Delphi process. A total of 30 participants from diverse stakeholder groups were invited to participate. The final voting process required participants to indicate (yes/no) whether the proposed COS should be regarded as the clinical evaluation set for ONFH using a secure interactive voting process. Although all votes were made independently and in private, the level of agreement was immediately communicated via the voting procedure. An agreement level of 70% was required for recommendation; 86.7% (26/30) of the participants agreed to the proposed COS, and consensus was reached as a result.

## Results

The COS of joint-preserving treatment for ONFH was developed through systematic reviews, modified Delphi surveys, and consensus meetings. Pain, ROM of hip flexion, walking distance, and stable rating of X-ray images constituted the COS. These indicators are recommended for assessing joint-preserving treatment for ONFH.

### Visual analogue scale (VAS)

**Fig. 1:**
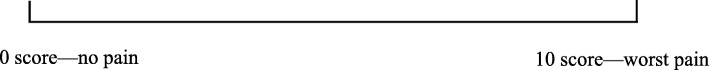
Schematic diagram of the visual analogue scale for pain intensity

The patient is asked to make a mark on a 100-mm horizontal line according to his or her degree of pain (Fig. 1). Zero (“0”) is selected when the patient is experiencing no pain, and the worst possible pain is assigned 10 points.

### ROM of hip flexion

**Fig. 2:**
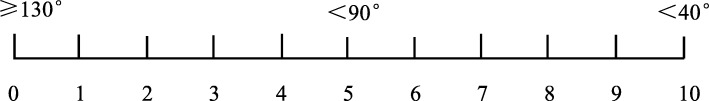
Schematic diagram of the ROM of hip flexion measurement

Hip flexion motion is assigned a score of 10 when the range is less than 40°, which indicates that the patient’s hip joint activity is severely limited, and the function is very poor; the score is 0 if the range is greater than or equal to 130°, which shows that the patient’s hip joint motion is normal, and function is good (Fig. 2). The higher the score, the greater the dysfunction; lower scores indicate better motion and function.

### Walking distance

**Fig. 3:**
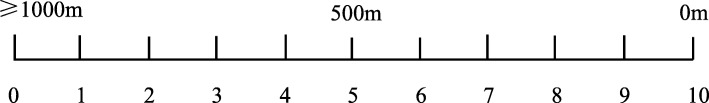
Schematic diagram of walking distance measurement

The patient is asked to make a mark on a horizontal line according to his or her maximum outdoor walking distance in daily life without support (Fig. 3). The highest score is 10 points, representing the shortest outdoor walking distance; this score indicates that the patient cannot walk outdoors by him- or herself or is confined to a wheelchair or to bed. A score of zero (“0”) point is assigned when the patient can walk more than 1000 m outdoors without any aids.

### Ratio of different indicators to the total score

The total clinical evaluation score (Table 4) is a weighted sum of the aforementioned three aspects with a ratio of 5:3:2 for the VAS, ROM of hip flexion, and walking distance. The highest score is 10, and the lowest score is 0. The higher the score, the more severe the disease is.

**Table 4:** Comprehensive clinical evaluation method

Outcomes	Score	Ratio	Final score	Total score
VAS	0~10	0.5	0~5	0~10
ROM of hip flexion	0~ 10	0.3	0~3	
Walking distance	0~ 10	0.2	0~2	

### Stable rating of X-ray images

We defined the stable rating of X-ray images as follows: the necrotic area of the femoral head had a similar or the same morphology before and after treatment, the necrotic area showed that bone density had increased, cystic size was reduced, and the sclerosis zone was blurred.

## Discussion

No published COS for joint-preserving treatment in ONFH effectiveness trials has been developed with key stakeholders using a standardized and transparent methodology. Based on the outcomes of assessments of joint-preserving treatment for ONFH extracted from the systematic review, the modified Delphi method and consensus meeting were used to collect and analyze the opinions of different stakeholders. These stakeholder opinions were then used to develop the basic COS that should be reported in clinical trials of joint-preserving treatment for ONFH.

Through three rounds of Delphi surveys and a consensus meeting, with participation by doctors and patients, a consensus opinion was reached. The resulting COS comprised clinical indicators and radiological indicators and combined subjective and objective evaluations. The clinical indicators included pain, ROM of hip flexion, and walking distance; the radiological indicators included stable rating of X-ray images.

The reasons for choosing pain as an indicator were as follows. First, pain is the most easily perceived symptom of orthopedic diseases and is the most important to relieve immediately. Therefore, pain assessment is one of the most important indicators for evaluating the effectiveness of interventions, and it is easier to reach a consensus regarding its importance [27–29]. Second, in HHS [4], which is widely used in clinical practice, pain accounts for 44 points out of 100 points. In the Charnley score [8], the degree of pain is one of three items used for evaluating hip joint function. Pain occupies a large proportion of the above evaluation criteria, which is sufficient to explain its importance.

Improving joint function and meeting the needs of daily life are the basic demands for the treatment of ONFH [30]. Many movements, including walking, climbing stairs, and wearing shoes and socks, are closely related to hip flexion. In clinical practice, a hip flexion examination is one of the physical examinations most commonly used by clinical orthopedic doctors to evaluate hip function [31]. If the degree of flexion tends to be normal, the degree of other movements is also likely to be good and can meet the needs of patients’ daily lives. In the original HHS [4], flexion accounted for 78 points out of the total score of 100.5, and joint activity accounted for 77.6%. Therefore, the HHS regards flexion as the most important evaluation index and gives it the maximum score. Among other scores for hip diseases, similar choices illustrate that flexion is the most important direction of activity. In the literature review [32, 33], many studies use hip joint flexion, hip extensor strength, and pain as independent research objects to analyze the impact of disease on joint function and have separately proposed the flexion rating in the HHS as a measure for determining change in the efficacy evaluation.

Walking distance represents the patient’s daily living ability [34]. If the patient walks well autonomously, it is sufficient to show that the patient can complete basic daily activities such as going up and down the stairs and taking busses. For this reason, walking distance is used as an independent evaluation indicator in many studies [35, 36].

The reasons for choosing X-ray imaging are as follows. First, when a patient has been clearly diagnosed with ONFH, X-ray is the most basic and important imaging examination. Anteroposterior and lateral radiography positions are simple and easy to repeat. Images taken at two different time periods are easy to compare. Comparisons between CT and MRI images are difficult due to the large number of layers and the difference in radiography positions [37]. Second, indicators for evaluating interventions can be divided into intermediate indicators and endpoint indicators. X-ray imaging is an objective indicator; although it is not an absolute endpoint indicator in the same was as artificial total hip arthroplasty, for example, it is an indicator that can explain objective changes in disease conditions and is biased towards the endpoint. Therefore, the COS selected the stable rating of X-ray images as the long-term outcome for efficacy evaluation.

The definition of COS is a minimum set of indicators that should be reported that has the characteristics of consensus and importance. Therefore, we comprehensively considered the subjective and objective indicators, their number and clinical operability, and selected three clinical indicators plus stable rating of X-ray images to evaluate joint-preserving treatment for ONFH. It is hoped that these indicators will always be reported in future hip preservation treatment effectiveness trials to enable the comparison of data among studies to provide high-quality evidence for clinical practice and to simultaneously achieve the goals of simplifying the experimental design and reducing the risk of bias. However, these indicators are not limited, and authors are encouraged to explore additional significant indicators to provide sufficient evidence for the improvement of the COS in the future. For example, the establishment of primary endpoint indicators is as meaningful as the establishment of intermediate indicators; although primary endpoint indicators were not established in this process of creating the COS due to the difficulty of observing them, the possibility that such indicators will be included in the future is not excluded.

The COS was established through a systematic literature review, Delphi surveys, and a consensus meeting. The four indicators of pain, ROM of hip flexion, walking distance, and stable rating of X-ray images were recommended.

## Conclusion

We established the COS for joint-preserving treatment of ONFH, which includes four indicators: pain, ROM of hip flexion, walking distance, and stable rating of X-ray images. We recommend the COS for the evaluation of hip preservation therapy for ONFH; we expect that these indicators will always be reported in future trials, and we encourage the authors to explore more meaningful outcome indicators. The current core outcome set will be verified and improved in future clinical research.

## Data Availability

The key data generated or analyzed during this study are included in this published article and the supplementary information files. Moreover, the datasets supporting the conclusions of the present study are available from the corresponding author on reasonable request.

## Abbreviations

COS: Core outcome set
HHS: Harris Hip Score
JOA: Japanese Orthopedic Association Score
ONFH: Osteonecrosis of the femoral head
RCT: Randomized controlled trial
ROM: Range of motion
THA: Total hip arthroplasty
VAS: Visual analogue scale
WOMAC: Western Ontario and McMaster Universities Osteoarthritis Index
